# Promoter Methylation of *QKI* as a Potential Specific Biomarker for Early Detection of Colorectal Cancer

**DOI:** 10.3389/fgene.2022.928150

**Published:** 2022-08-09

**Authors:** Lei Zhang, Dapeng Li, Lijing Gao, Jinming Fu, Simin Sun, Hao Huang, Ding Zhang, Chenyang Jia, Ting Zheng, Binbin Cui, Yanlong Liu, Yashuang Zhao

**Affiliations:** ^1^ Department of Epidemiology, College of Public Health, Harbin Medical University, Harbin, China; ^2^ Department of Colorectal Surgery, Harbin Medical University Cancer Hospital, Harbin Medical University, Harbin, China

**Keywords:** colorectal cancer, diagnostic, *QKI*, DNA methylation, biomarker

## Abstract

Early and specific detection of cancer provides an opportunity for appropriate treatment. Although studies have suggested that *QKI* is a tumor suppressor gene, no studies have evaluated the diagnostic utility of *QKI* methylation in colorectal cancer (CRC). Here, we evaluated the methylation status of *QKI* by integrating the methylation data of tissues and cell lines of multiple cancer types. The diagnostic performance of *QKI* was analyzed in the discovery dataset from the TCGA CRC 450K array (*n* = 440) and tested in the test sets (*n* = 845) from the GEO. The methylation level of *QKI* was further validated in our independent dataset (*n* = 388) using targeted bisulfite sequencing. All detected CpG sites in the *QKI* promoter showed CRC-specific hypermethylation in 31 types of tumor tissues. In the discovery dataset, six consecutive CpG sites achieved high diagnostic performances, with AUCs ranging from 0.821 to 0.930. In the test set, a region (chr6: 163,834,452–163,834,924) including four consecutive CpG sites had robust diagnostic ability in distinguishing CRC and adenoma from normal samples. In the validation dataset, similar robust results were observed in both early- and advanced-stage CRC patients. In addition, *QKI* exhibited hypermethylation in the cfDNA of patients with CRC (n = 14). Collectively, the *QKI* promoter is a CRC-specific methylation biomarker and holds great promise for improving the diagnosis using minimally invasive biopsy.

## Introduction

With an estimated 1.9 million new cases occurring in 2020, colorectal cancer (CRC) is the second most common cause of cancer-related death worldwide (Sung et al., 2021). CRC occurs via a multistep process of transition from benign neoplasm to overt adenocarcinoma that takes 10–15 years, which provides a window for cancer intervention and prevention (Vogelstein et al., 1988; Arber and Levin, 2005). With the current treatment options, the 5-year survival rate of patients with stage I or II is more than 80%. However, less than half of CRC cases are detected at early stages, and the early detection of CRC remains a challenge (Miller et al., 2019).

DNA methylation is a heritable epigenetic modification that modulates gene expression without changing the underlying DNA sequence (Bird, 2002). Aberrant hypermethylation of tumor suppressor genes occurs early in tumorigenesis, making it a promising biomarker for cancers, including CRC (Irizarry et al., 2009; Baylin and Jones, 2011; 2016). Meanwhile, there are several potential advantages of assessing methylation over tumor somatic mutations, including geometrical increases in the number of detectable sites, and the methylation patterns often reflect the epigenetic origin of specific cancers to reveal the origin of the unknown primary cancers (Chen et al., 2016; Moran et al., 2016).

In a liquid biopsy, circulating cell-free DNA (cfDNA) has emerged as a key biomarker for early diagnoses (Hao et al., 2017; Jin et al., 2021). Genetic or epigenetic changes can distinguish circulating tumor DNA (ctDNA) released from circulating tumor cells (CTCs) or primary tumor tissue from cfDNA derived from leukocytes (Schwarzenbach et al., 2011; Moss et al., 2018). For instance, the blood-based test of *SEPT9* methylation has been approved by the FDA for CRC screening (Lamb and Dhillon, 2017). However, this test showed varied performance in different studies and a high positive rate for other cancer types (Grützmann et al., 2008; Lee et al., 2013; Schrock et al., 2017; Jiao et al., 2019; Petit et al., 2019). Therefore, tests for cfDNA can be improved if they are combined with cancer-specific methylation patterns.

The RNA-binding protein quaking (*QKI*), which is a member of the signal transduction and activator of RNA metabolism (STAR) family (Vernet and Artzt, 1997), has been proven to affect pre-mRNA splicing, mRNA turnover, and translation (Saccomanno et al., 1999; Wu et al., 2002; Larocque et al., 2005). The function of *QKI* was first glimpsed in brain tumors, suggesting that it may have a role in tumor suppression (Li et al., 2002). Furthermore, the expression of *QKI* was greatly reduced or absent in the tissue of patients with CRC, and the change was accomplished at least partially by DNA hypermethylation (Yang et al., 2010; Iwata et al., 2017). A long non-coding RNA (lncRNA) named *CAHM* (chr6: 163,834,097–163,834,982), located adjacent to the *QKI* gene, has been demonstrated to be specifically hypermethylated in CRC and adenoma, with a low methylation level in the tissue of lung, prostate, and breast cancers (Pedersen et al., 2014). Given the fuzzy boundary of *QKI* and *CAHM*, the methylation of the *QKI* gene in CRC is worth exploring.

By integrating large-scale genome-wide methylation data of multiple cancer types from TCGA, we discovered that the *QKI* promoter was specifically hypermethylated in the CRC tissues but not in other cancer types or adjacent normal tissues. Although studies have found that *QKI* is hypermethylated in colorectal cancer tissues compared with normal tissues, whether hypermethylation of *QKI* is a promising molecular marker for early detection of CRC has not been investigated. Therefore, we further validated the potential diagnostic performance of *QKI* methylation for CRC in tissues, cell lines, and blood from public and in-house data.

## Materials and Methods

### Public Data Source

Publicly available datasets were obtained from the TCGA and GEO. The Illumina 450K methylation array of 31 types of cancer, including CRC, was downloaded from UCSC Xena^1^. Meanwhile, RNA-Seq gene expression data of CRC, as in log2 (x + 1) transformed RSEM-normalized count was obtained from UCSC Xena. The 450K array of cell lines of 29 different tissues was obtained from GSE68379 (Iorio et al., 2016). Whole-blood DNA methylation profiles from healthy donors were generated in GSE40279 (Hannum et al., 2013). Moreover, two independent test sets, A and B, were established using the 450K array from GEO, including GSE42752 (Naumov et al., 2013), GSE48684 (Luo et al., 2014), GSE101764 (Barrow et al., 2017), GSE131013 (Díez-Villanueva et al., 2020), GSE77954 (Qu et al., 2016), GSE129364 (Fiedler et al., 2019), and GSE139404 (Fan et al., 2020). An additional methylation dataset array was obtained from GSE122126, which provided the methylation profile of three cfDNA samples from CRC and four samples from healthy controls with the Illumina EPIC (Moss et al., 2018). The methylation level of the CpG sites was represented as beta values, which is a ratio of intensities between the methylated (M) allele and the sum of methylated (M) and unmethylated (U) alleles. The beta values are continuous and range from 0 (unmethylated) to 1 (completely methylated). Details of the included subjects are presented in Table 1. The genome information of CpG sites of the *QKI* gene in the 450K array is shown in Supplementary Table S1, and the CpG sites with the missing value above 10% in the TCGA CRC dataset were excluded from further analysis.

**TABLE 1:** The basic information of datasets used in the current study.

Dataset source	Sample source	Sample type	Platform	Number of samples
Discovery dataset (*n* = 11,292)				
TCGA	CRC	Tissue	450K array	Normal = 45, Tumor = 395
TCGA	30 cancer types	Tissue	450K array	Normal = 704, Tumor = 8535
GEO	GSE68379	Cell lines	450K array	Tumor = 957
GEO	GSE40279	Whole blood	450K array	Normal = 656
Test set (*n* = 845)				
Test set A	GSE42752	Tissue	450K array	Normal = 41, CRC = 22
	GSE48684^†^	Tissue	450K array	Normal = 41, CRC = 64
	GSE77954^†^	Tissue	450K array	Normal = 11, CRC = 13
	GSE101764	Tissue	450K array	Normal = 149, CRC = 110
	GSE131013	Tissue	450K array	Normal = 144, CRC = 96
Test set B	GSE48684^†^	Tissue	450K array	Normal = 41, Adenoma = 42
	GSE77954^†^	Tissue	450K array	Normal = 11, Adenoma = 12
	GSE129364	Tissue	450K array	Normal = 3, Adenoma = 30
	GSE139404	Tissue	450K array	Normal = 20, Adenoma = 40
Test set C	GSE122126	cfDNA	EPIC array	Normal = 4, CRC = 3
Validation dataset (*n* = 388)				
	In-house study	Tissue	Targeted bisulfite sequencing	Normal = 24, Polyps = 9, Adenoma = 8, CRC = 275
	In-house study	WBC	Targeted bisulfite sequencing	Normal = 29, CRC = 29
	In-house study	cfDNA	Targeted bisulfite sequencing	Normal = 5, CRC = 9

^†^The normal samples in GSE48684 and GSE77954 were shared in both test sets A and B. CRC, colorectal cancer; TCGA, The Cancer Genome Atlas; GEO, Gene Expression Omnibus; cfDNA, cell-free DNA; WBC: white blood cell.

### Patients and Sample Collection

In total, 316 fresh frozen tissue samples were collected, including 275 CRC tissues, 24 adjacent normal tissues, eight adenoma tissues, and nine hyperplastic polyp tissues. We also collected 29 white blood cell (WBC) samples from CRC patients and 29 WBC samples from healthy controls. Blood specimens of nine patients with CRC were collected in ardent cell-free DNA BCT before surgical and neoadjuvant chemotherapy, as well as five healthy normal individuals. The plasma was separated from the blood (8–10 ml) via double centrifugation (3,000 rpm for 10 min at RT, and then, 16,000 g at 4°C for another 10 min) within 4 h after the blood was drawn and stored at −80°C until cfDNA isolation. These samples were collected at the Third Affiliated Hospital of Harbin Medical University. All patients with CRC or precancerous lesions were newly diagnosed by postoperative pathology or colonoscopy pathology and underwent surgery without neoadjuvant chemotherapy. Healthy controls were free from a history of malignancy, and without any benign or malignant diseases confirmed by colonoscopy. Demographic and clinical information including age, gender, tumor location, and tumor stage were obtained from medical records and pathological reports (Supplementary Table S2). All procedures performed in studies involving human participants complied with the 1964 Helsinki Declaration and its later amendments or comparable ethical standards.

### Sample Preparation and Targeted Methylation Sequencing

Tissue and WBC genomic DNA (gDNA) were extracted using the phenol-chloroform method and QIAamp DNA Mini kit (Qiagen, Cat# 51306). cfDNA was isolated from 4–5 ml plasma using the QIAamp Circulating Nucleic Acid Kit (Qiagen, Cat# 55114) following the manufacturer’s protocol. The concentration of cfDNA was measured using the Qubit™ dsDNA HS Assay Kit (Thermo Fisher Scientific, Cat# Q32851). All samples were bisulfite-converted using the EZ DNA Methylation-Gold™ Kit (Zymo Research, Cat# D5006) according to the instruction manual. Targeted bisulfite sequencing is a next-generation sequencing-based technology for the quantization of the methylation of targeted regions at a base-specific level. We designed three regions to measure the methylation level of *QKI* in tissues and WBCs, and a short overlapping region in cfDNA (Supplementary Table S3). Multiplex PCR was performed first and amplified further using indexed primers, and target capture libraries were sequenced on Illumina’s MiSeq with a 2 × 150 bp paired-end mode (Genesky Biotechnologies Inc.). In data analysis, samples with bisulfite conversion rate <98%, or average coverage less than ×10 were filtered out.

### Statistical Analyses

The Student’s t-test was used to compare the differences in the methylation level of *QKI* between the two groups. The Spearman correlation method was used to assess the co-methylation of adjacent CpG sites and the correlation between methylation and expression (R package ‘corrplot’, version 0.84). The area under the curve (AUC) obtained from a receiver operating characteristic (ROC) curve analysis was used to test the diagnostic accuracy, and the optimal cut-off value for the CpG site of *QKI* was selected at the maximal Youden index. AUC values between subgroups were compared using the DeLong method (R package ‘pROC’, version 1.17.0.1). A trend test of the methylation level of *QKI* in different neoplastic progression states was performed with a linear regression model. We also used R packages such as ggplot2 (version 3.3.3), ggsignif (version 0.6.1), tidyverse (version 1.3.0), pheatmap (version 1.0.12), and survival (version 3.2-7). All statistical analyses were conducted using R software (version 4.0.4). A two-sided *p* < 0.05 was considered statistically significant. The human genome version GRCh37/hg19 was used as the reference for genomic coordinates.

## Results

### Tissue-specific Methylation of the Quaking Gene in Colorectal Cancer

**FIGURE 1:**
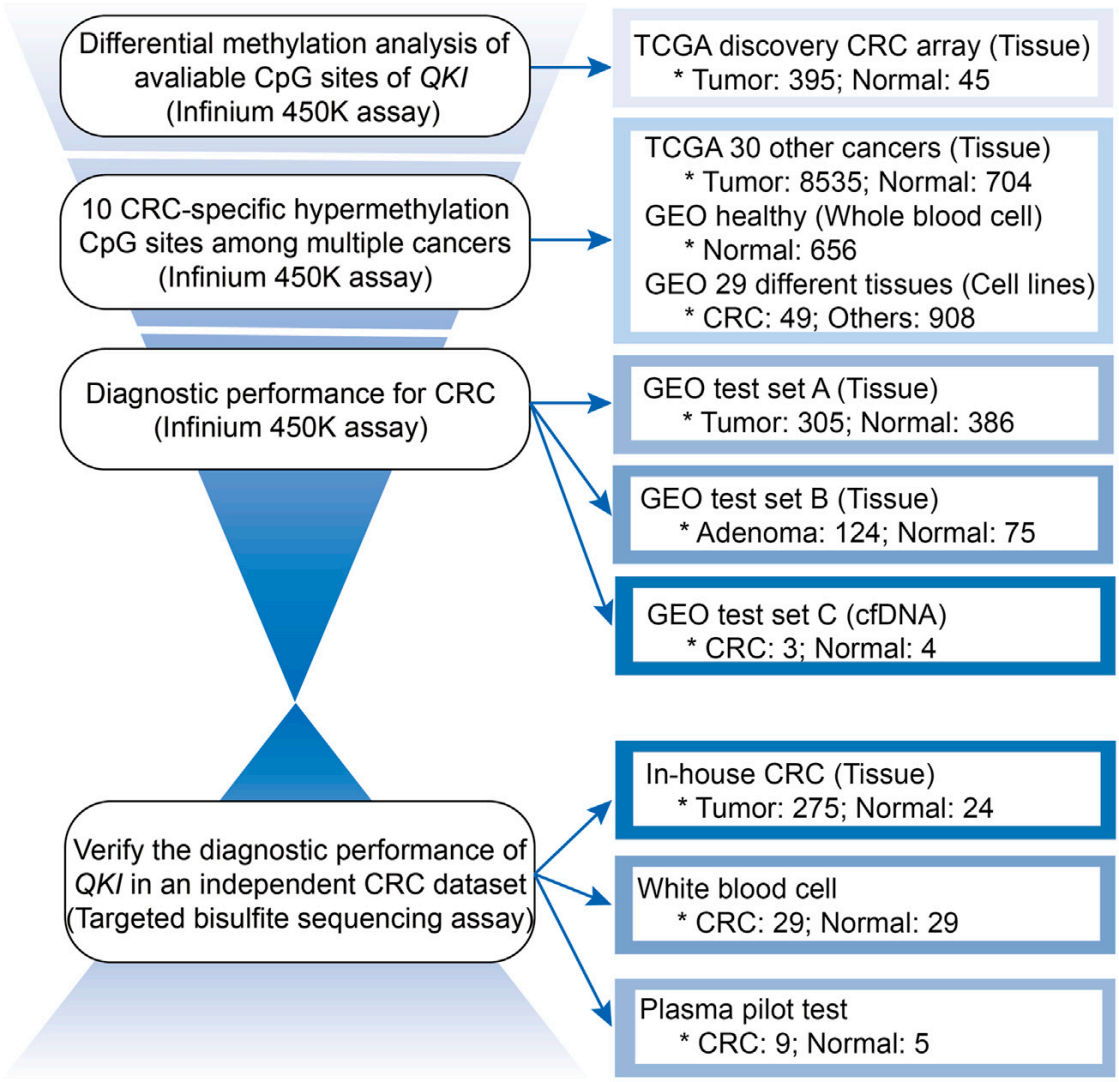
Workflow for analyzing the methylation level of the *QKI* gene in multiple datasets.

**FIGURE 2:**
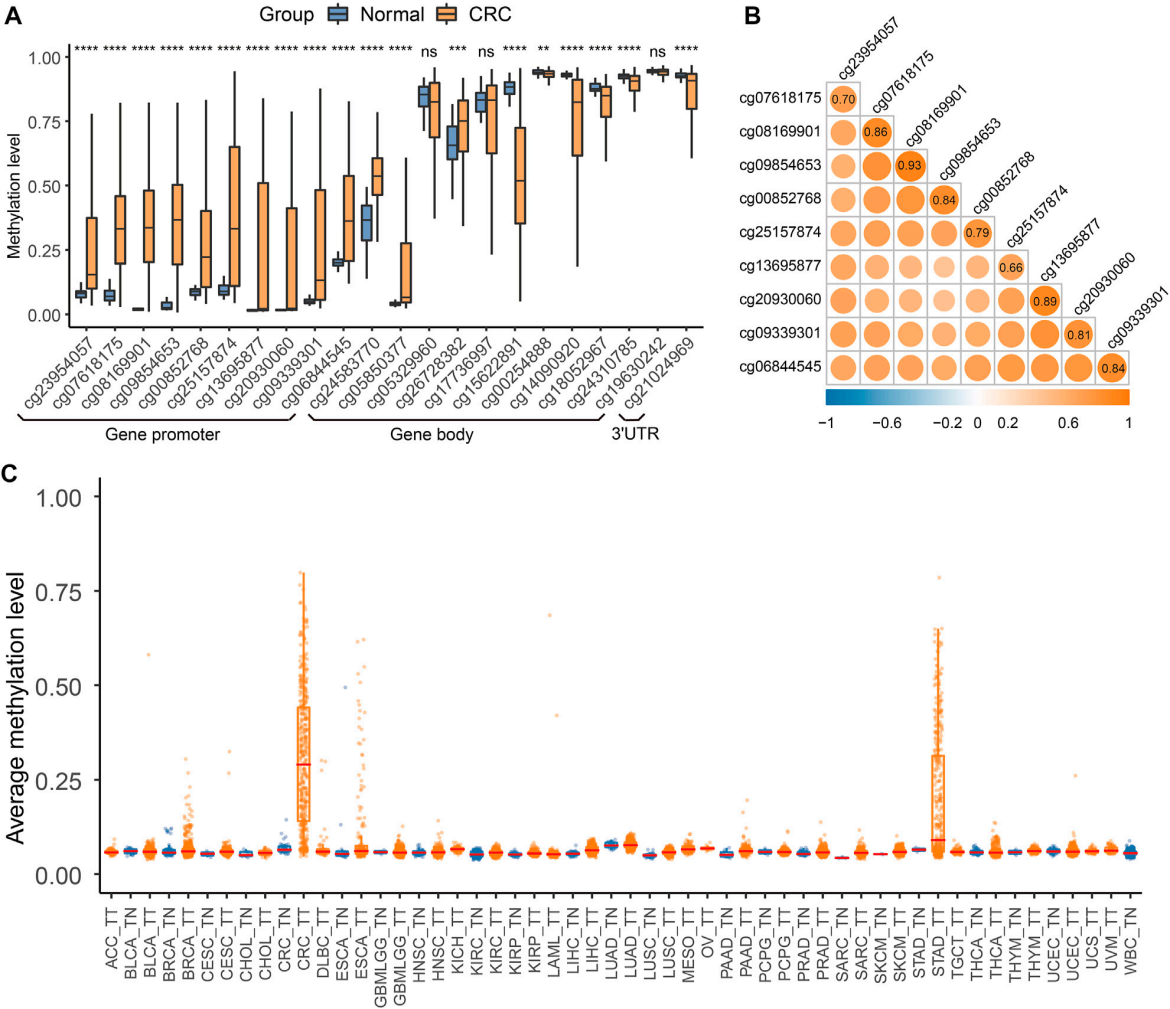
*QKI* methylation analysis in the discovery dataset. **(A)** Differential methylation analysis for 22 CpG sites of the *QKI* gene between colorectal cancer tissues and normal tissues in the TCGA CRC dataset. Gene promoter was defined as the region containing TSS200, TSS1500, 5′UTR, and the first exon. Symbols indicating statistical significance: NS, *p* > 0.05; *, *p* ≤ 0.05; **, *p* ≤ 0.01; ***, *p* ≤ 0.001; ****, *p* ≤ 0.0001. **(B)** Co-methylation analysis of 10 CpG sites in the promoter region of *QKI*. **(C)** Boxplot of the average methylation level of the *QKI* promoter in multiple cancers.

The workflow is described in Figure 1. There were 22 available CpG sites of *QKI* after removing three CpG sites with missing values above 10% in the TCGA CRC dataset. The methylation level of 10 CpG sites in the *QKI* promoter was significantly higher in CRC than those in normal tissues, and the neighboring CpG sites were highly correlated with each other (termed as co-methylation, Figures 2A,B, Supplementary Table S4). In 391 CRC samples with both methylation and expression data, *QKI* promotor methylation was negatively correlated with the gene expression (average r = −0.331, *p* < 0.001; Supplementary Figure S1).

We first assessed whether *QKI* methylation was a tissue-specific alteration of CRC by comparing the genome-wide methylation data of tumor tissues (*n* = 8930) and adjacent normal tissues (*n* = 749) of 31 cancer types from TCGA. We found that 10 CpG sites in the *QKI* promoter were specifically hypermethylated in CRC tissues but not in tumor tissues from 30 other cancer types or normal tissues (Figure 2C). Given that peripheral blood leukocytes are the main source of cfDNA, methylation levels of the *QKI* promotor were relatively low in whole-blood samples from healthy individuals (*n* = 656, Supplementary Figure S2). Moreover, the *QKI* promoter was specifically hypermethylated in cell lines of CRC (*n* = 49) compared with cell lines of other cancer cell lines (*n* = 908, Supplementary Figure S3). In summary, *QKI* promoter methylation is a tissue-specific marker of CRC that can distinguish CRC tissues from the tumor or normal tissues of other cancer types.

### The Diagnostic Performance of Methylation of Quaking Promoter in Colorectal Cancer and Adenoma From the TCGA and Test Sets

**FIGURE 3:**
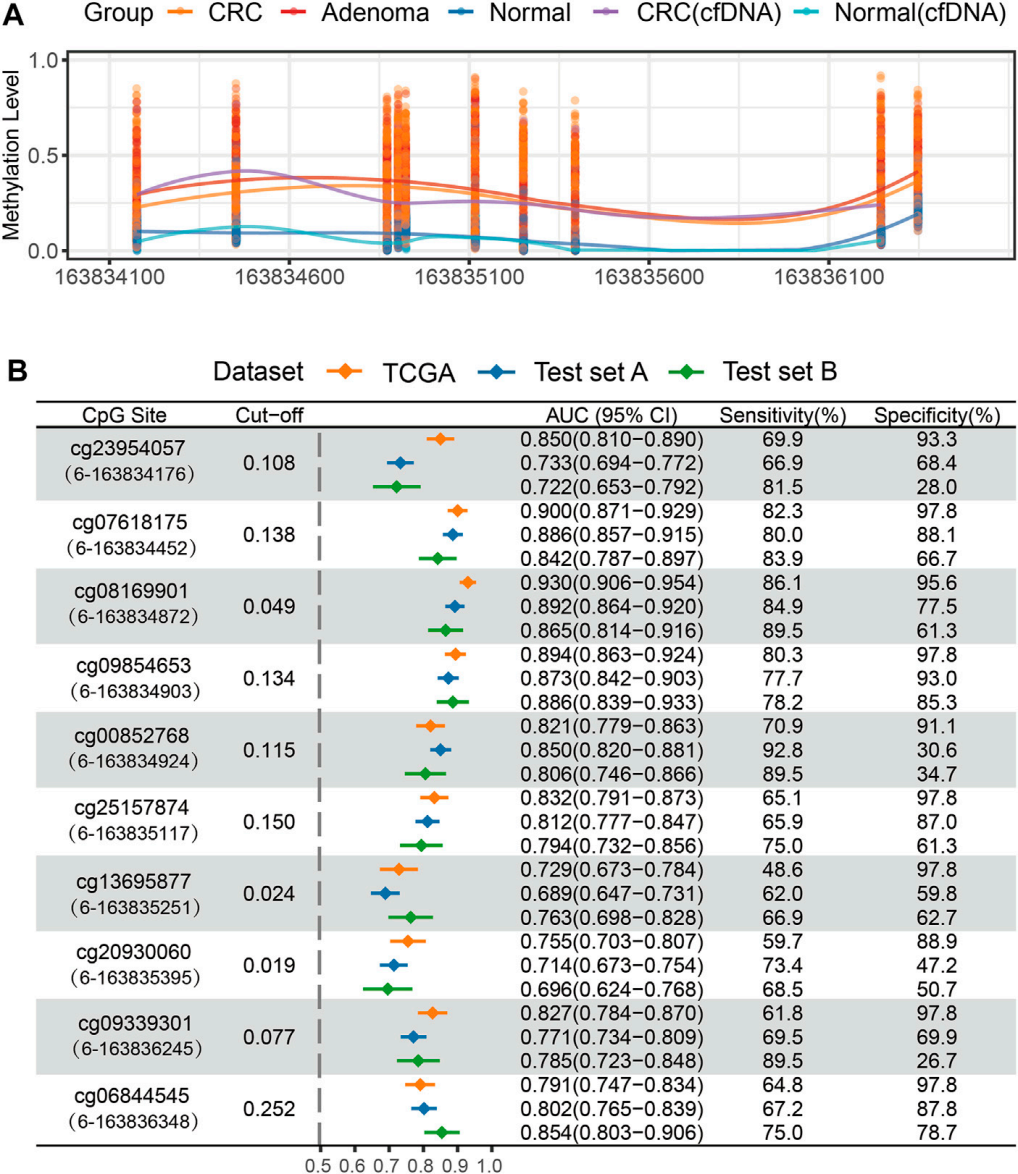
Methylation analysis of *QKI* promoter in the test set. **(A)** The methylation level of 10 CpG sites of the *QKI* promoter in the test set. **(B)** ROC curve analysis of 10 CpG sites of the *QKI* promoter for discriminating CRC or adenoma tissues from normal tissues in the discovery and test dataset. The content in brackets denotes the “chromosome”-“coordinate” of each CpG site.

We evaluated the diagnostic performance of 10 CpG sites in distinguishing CRC or adenoma from normal tissues. The six consecutive CpG sites (cg23954057, cg07618175, cg08169901, cg09854653, cg00852768, and cg25157874) showed high discriminating ability, with AUC values ranging from 0.821 to 0.930 in the TCGA CRC dataset. Furthermore, an elevated methylation level of 10 CpG sites in the *QKI* promoter was found in the CRC of the test set A, and five of six CpG sites had a stable performance in distinguishing 305 CRC patients from 386 normal tissues (average AUC = 0.863). The average sensitivity and specificity of the five CpG sites were 80.3 and 75.2% (Figure 3), respectively. Next, we assessed the diagnostic performance of the 10 CpG sites to distinguish 124 adenomas from 75 normal tissues in test set B. The 10 CpG sites were significantly hypermethylated in adenomas. Four CpG sites (cg07618175, cg08169901, cg09854653, and cg00852768) achieved high diagnostic ability, with AUCs >0.800 (Figure 3). Collectively, the region including four consecutive CpG sites (chr6: 163,834,452–163,834,924) was identified as a candidate marker for diagnosing CRC and its precancerous lesions.

### Quaking Methylation Status in the Cell-Free DNA Sample of Colorectal Cancer

In addition, we evaluated the methylation status of the *QKI* promoter in cfDNA samples from three CRC patients and four healthy controls. Hypermethylation of eight available CpG sites was observed in at least one cfDNA sample from CRC, but not in cfDNA samples from healthy individuals (Figure 3A, Supplementary Figure S4).

### Diagnostic Performance of Quaking Methylation in Colorectal Cancer Tissues From the Validation Dataset

**FIGURE 4:**
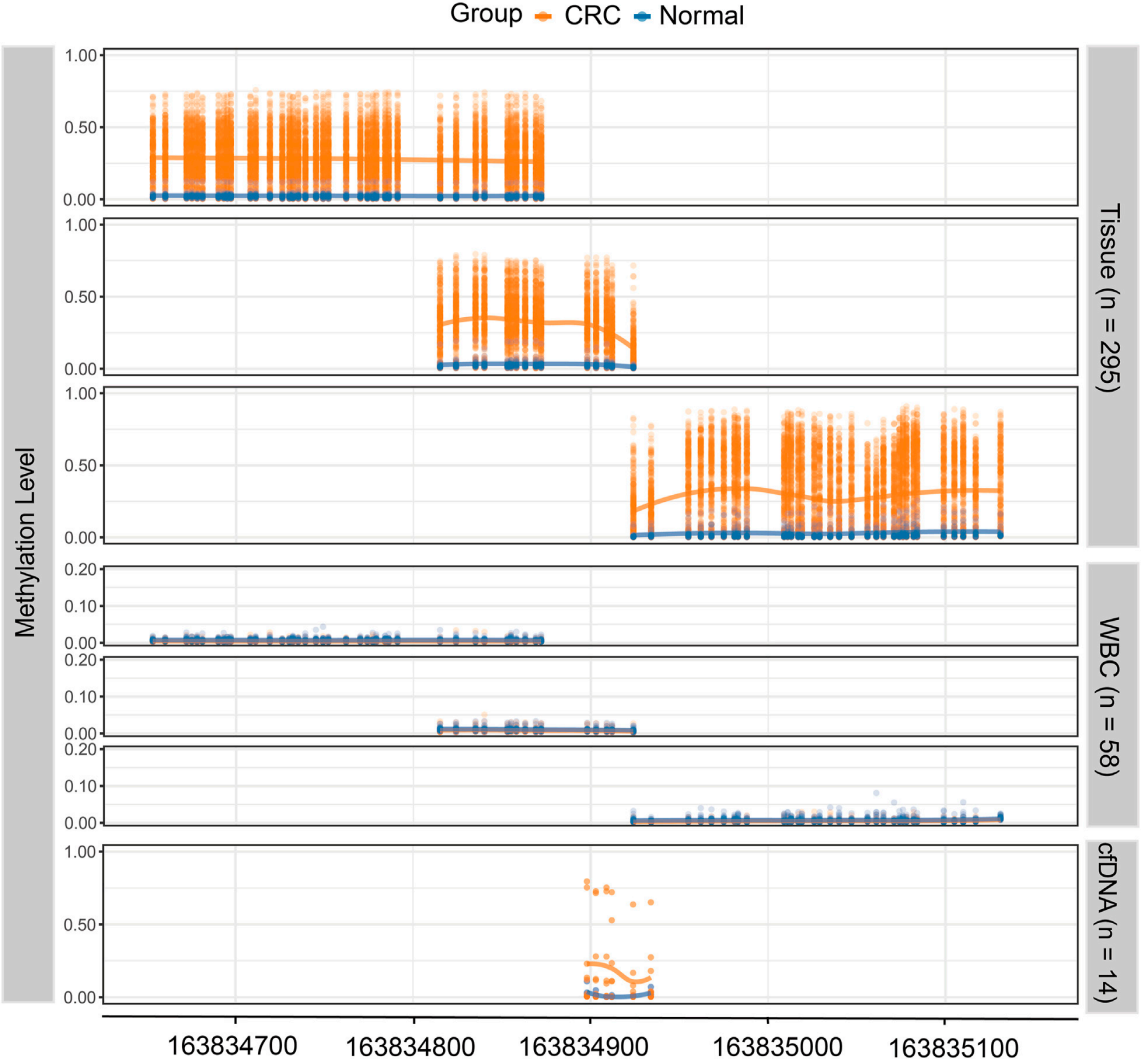
The methylation level of the *QKI* target region in the validation dataset. The top three panels show the methylation level of 271 CRC tissues and 24 normal tissues, where each dot represents one CpG site for each sample. The middle three panels show the methylation level of white blood cells from CRC patients (*n* = 29) and healthy controls (*n* = 29). The lowest panel shows the methylation level of cell-free DNA samples from CRC patients (*n* = 9) and healthy controls (*n* = 5).

**FIGURE 5:**
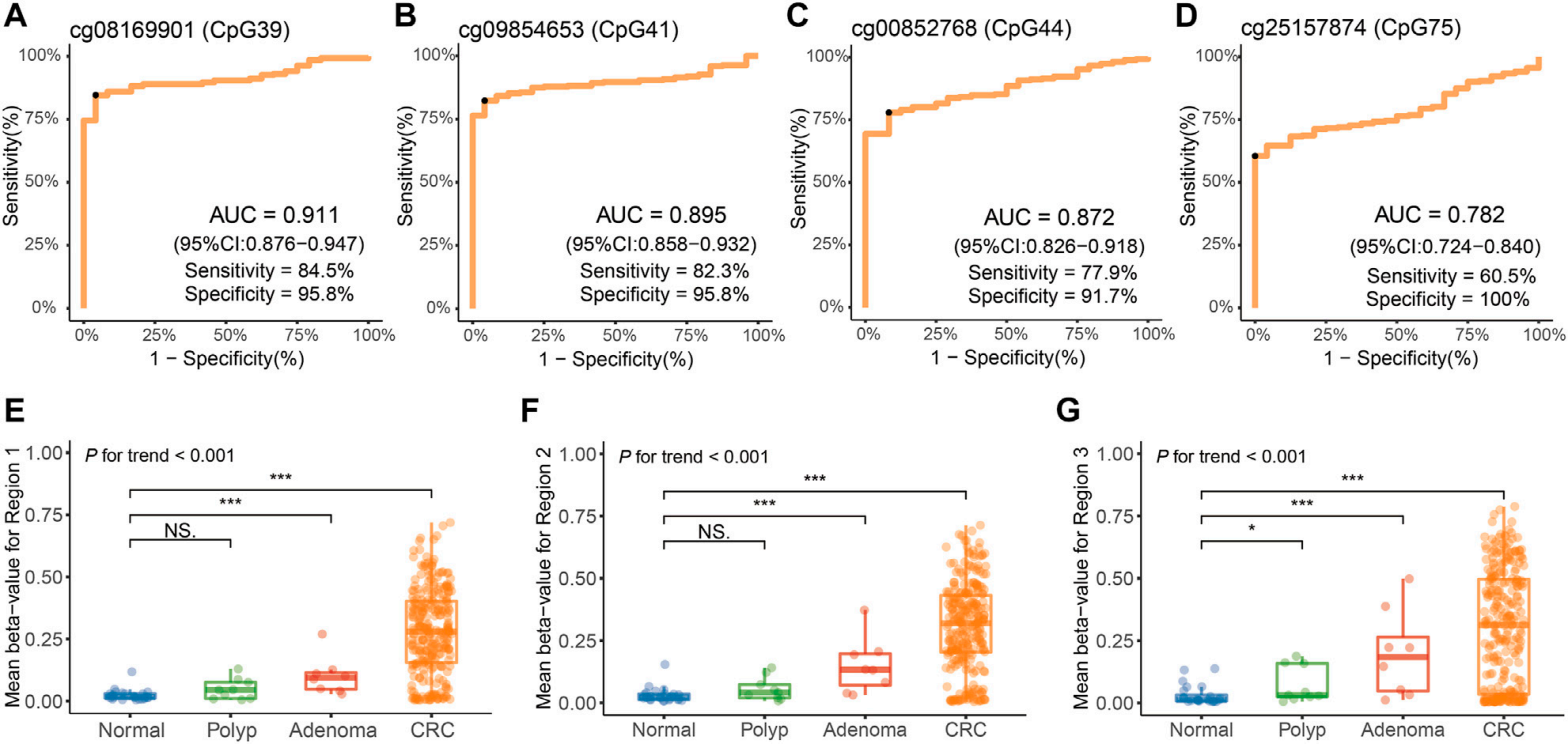
Methylation analysis of the *QKI* promoter in the validation dataset. **(A–D)** Diagnostic performance of four target CpG sites for distinguishing CRC from adjacent normal tissues. The black point indicates the optimal cut-off value for each CpG. **(E–G)** Average methylation levels of three regions of the *QKI* promoter at different disease stages during neoplastic progression of colorectal cancer tissues. Symbols indicating statistical significance: NS, *p* > 0.05; *, *p* ≤ 0.05; **, *p* ≤ 0.01; ***, *p* ≤ 0.001; ****, *p* ≤ 0.0001.

Based on the genomic coordinates of the *QKI* promoter in the discovery dataset, we designed three amplification regions (chr6: 163,834,632–163,835,160; Supplementary Tables S5–S7) for targeted bisulfite sequencing. A total of 76 CpG sites were detected in the three target regions, covering four CpG sites in the 450K array. Three samples with bisulfite conversion rate <98% and one sample with average coverage less than 10× were excluded. Differential methylation analysis showed that all 76 CpG sites in the target regions were hypermethylated in 271 CRC samples compared to 24 normal tissues (Figure 4). The cg08169901 (CpG39) was detected repeatedly in region 1 and region 2, and the AUC values were 0.896 and 0.911, respectively. We preferred the detection result of region 2 since it was located in the middle of target region 2, and its AUC was slightly lower than that of the discovery dataset (AUC = 0.930). The AUC value of cg09854653 (CpG41) was consistent with the discovery dataset (0.895 vs. 0.894). The cg00852768 (CpG44) appeared at the boundary of both target 2 and target 3, with AUC values (0.872 and 0.867, respectively) similar to those of the discovery dataset (AUC = 0.821). The AUC value of cg25157874 (CpG75) was lower than that of the TCGA dataset (0.782 vs. 0.830). The sensitivity of the four target CpG sites was 84.5, 82.3, 77.9, and 60.5%, with corresponding specificities of 95.8, 95.8, 91.7, and 100%, respectively (Figures 5A–D, Supplementary Tables S5–S7).

Furthermore, we evaluated the diagnostic performance of the target CpG sites in different subgroups, including age, gender, tumor location, and tumor stage (Table 2). AUC values of the cg08169901 (CpG39), cg09854653 (CpG41), and cg00852768 (CpG44) were consistent among the different subgroups. However, cg25157874 (CpG75) had a higher AUC value when the tumor was located at the rectum (0.710 vs. 0.822, *p* = 0.043). Indeed, four CpG sites had consistent diagnostic performance in early-stage (stage I/II) and advanced-stage (stage III/IV) patients with CRC. In addition, the remaining CpG sites in region 1 (Supplementary Table S5) and region 2 (Supplementary Table S6) had similar AUC values ranging from 0.885 to 0.918, while the AUC values of region 3 (Supplementary Table S7) were slightly lower, having a range from 0.757 to 0.861.

**TABLE 2:** The AUC values of four CpG sites in the subgroup of different clinicopathological factors in the validation dataset.

Characteristics	cg08169901 (CpG39)		cg09854653 (CpG41)		cg00852768 (CpG44)		cg25157874 (CpG75)	
	AUC (95% CI)	*p*-value	AUC (95% CI)	*p*-value	AUC (95% CI)	*p*-value	AUC (95% CI)	*p*-value
Overall (n = 271)	0.911 (0.876–0.947)		0.895 (0.858–0.932)		0.872 (0.826–0.918)		0.782 (0.724–0.840)	
Subgroup								
Age								
≥60 (*n* = 128)	0.925 (0.884–0.966)	0.418	0.916 (0.873–0.960)	0.239	0.905 (0.857–0.953)	0.110	0.824 (0.760–0.889)	0.115
<60 (*n* = 143)	0.899 (0.852–0.947)		0.876 (0.824–0.927)		0.842 (0.782–0.902)		0.745 (0.670–0.819)	
Gender								
Male (*n* = 169)	0.907 (0.864–0.949)	0.704	0.885 (0.838–0.932)	0.464	0.848 (0.792–0.905)	0.102	0.765 (0.696–0.833)	0.366
Female (*n* = 102)	0.919 (0.872–0.966)		0.911 (0.860–0.962)		0.911 (0.862–0.960)		0.811 (0.738–0.883)	
Tumor location								
Colon (*n* = 96)	0.877 (0.817–0.938)	0.139	0.852 (0.787–0.918)	0.093	0.836 (0.765–0.904)	0.194	0.710 (0.618–0.801)	**0.043**
Rectum (*n* = 175)	0.930 (0.895–0.966)		0.918 (0.880–0.957)		0.892 (0.846–0.938)		0.822 (0.764–0.880)	
Tumor stage^†^								
I–II (*n* = 142)	0.896 (0.849–0.944)	0.366	0.882 (0.831–0.932)	0.485	0.865 (0.810–0.921)	0.783	0.770 (0.698–0.842)	0.638
III–IV (*n* = 125)	0.926 (0.884–0.967)		0.906 (0.859–0.953)		0.876 (0.821–0.932)		0.794 (0.725–0.864)	

The bold value emphasize that P < 0.05. ^†^There are four colorectal cancer patients without tumor stage. AUC, area under the curve.

### Quaking Methylation Status in Colorectal Precancerous Lesions From the Validation Dataset

Furthermore, we detected the methylation level of the *QKI* promoter in eight adenoma tissues and nine hyperplastic polyp tissues. In three target regions, the average methylation of adenoma (median beta-value of 0.094 for target region 1, 0.133 for target region 2, and 0.184 for target region 3) was significantly higher than that in normal tissues (median beta-value of 0.016 for target region 1, 0.022 for target region 2, and 0.012 for target region 3). No statistically significant difference was observed between polyps and normal tissues in target region 1 and region 2. There was an obviously increasing trend from polyps to adenomas to CRC (*P* for trend <0.001, Figures 5E–G).

### Quaking Methylation Status in White Blood Cells From the Validation Dataset

Methylation levels of three target regions were detected in WBC samples from 29 CRC patients and 29 healthy controls. The median methylation level of the three target regions was 0.005 for CRC (range, 0.003–0.012) and 0.008 for healthy controls (range, 0.004–0.014; Figure 4, Supplementary Tables S8–S10). The low level of methylation in WBCs from both CRC and healthy controls is unlikely to cause confusion in the detection of the cfDNA methylation level.

### Plasma Pilot Study

There are six CpG sites in the target region, covering cg09854653 (CpG41) and cg00852768 (CpG44, Supplementary Table S11). As shown in Figure 4, the methylation level of six CpG sites was significantly higher in nine cfDNA samples of CRC (range, 0.106–0.230) compared to five healthy controls (range, 0.003–0.036). Potentially, the methylation status of six CpG sites in this region was extracted to form methylation haplotypes, which accounted for a higher proportion of cfDNA from CRC patients than healthy controls (Supplementary Figure S5). In short, the methylation of the *QKI* promoter is promising as a specific marker for the non-invasive detection of CRC.

## Discussion

In this study, we demonstrated that the *QKI* promoter is a tissue-specific methylation marker for CRC by integrating large-scale public methylation datasets. A region including four consecutive CpG sites (chr6: 163,834,452–163,834,924) has excellent diagnostic performance in distinguishing CRC and adenoma tissues from normal tissues, which was further validated by a PCR-based method. As expected, methylation of the *QKI* promoter remains robust in diagnosing CRC, even in the early stages of cancer. A gradually increasing methylation level was observed during the tumorigenesis of CRC. The pilot cfDNA test suggested that methylation of the *QKI* promoter has the potential to be a non-invasive diagnostic marker for CRC.

An ideal biomarker of CRC should identify the tissue of origin of the tumor in clinical practice, and have considerable ability to diagnose colorectal adenoma. Previous studies have demonstrated that the combination of cancer-specific DNA methylation patterns and ctDNA can achieve highly specific detection in more than 38 cancer types, and detect asymptomatic individuals who were diagnosed later (Moran et al., 2016; Chen et al., 2020; Liu et al., 2020). Integrating large-scale datasets of multiple cancer types, our genome-wide methylation analysis identified that *QKI* was specifically hypermethylated in CRC tissues but not in other cancer types. We found that *QKI* methylation can distinguish both CRC and colorectal adenoma tissues from adjacent normal tissues. Collectively, methylation of the *QKI* promoter is a tissue-specific methylation alteration of CRC and it occurs early in the process of CRC formation.

Although previous genome-wide methylation studies have achieved high accuracy in diagnosing multiple types of cancers, the single-gene approach is more suitable for screening CRC in large-scale populations. Therefore, we validated hypermethylation of the *QKI* promotor in CRC tissues using a cost-effective PCR-based technology, with results consistent with the genome-wide methylation data. In addition, our analysis suggested that *QKI* methylation in cfDNA of CRC patients was not derived from DNA released from WBCs. Moreover, our plasma pilot study suggested that the *QKI* gene was hypermethylated in cfDNA samples from CRC patients. Both the high-throughput detection method and single-gene approach suggested that methylation of the *QKI* promoter was a candidate early diagnostic marker of CRC.

We extended the analysis of *QKI* methylation in cfDNA to the analysis of the methylated haplotypes. Early sequencing studies have indicated that adjacent CpG sites share similar methylation patterns (Eckhardt et al., 2006). Locally disordered methylation has also been observed, which indicates that methylation heterogeneity is primarily from variability within DNA fragments (Landau et al., 2014). Therefore, some studies have extended the theoretical framework of linkage disequilibrium (Slatkin, 2008) to CpG co-methylation analysis to annotate these methylation blocks as methylation haplotypes with a unique set of genomic features (Shoemaker et al., 2010). Studies have shown that methylated haplotypes show high accuracy in predicting the tissue of origin and the cancer status in cfDNA, whether it is built on 450K sparse genome coverage or the whole-genome sequencing analysis (Lehmann-Werman et al., 2016; Guo et al., 2017). Therefore, methylation haplotype detection may be more suitable for tumor-derived ctDNA without a clear source. As shown in our results, the methylated haplotype of cfDNA provides a reference for the primer design of PCR-based methods, and we hypothesize that identifying different haplotypes of patients at different stages can achieve an accurate diagnosis.

Although Yang et al. (2010) and Novikov et al. (2011) have provided evidence that *QKI* acts as a tumor suppressor gene and the down-regulation of *QKI* expression may be involved in CRC onset and progression, no study has assessed the diagnostic utility of *QKI* methylation in CRC. Particularly, a PCR-based region (chr6: 163,834,393–163,834,455) of *CAHM* demonstrated frequent methylation, with a positive rate of 81% (17/21) in adenomas and 71% (56/79) in CRC tissues. Indeed, increased methylation of *CAHM* was also found in the plasma DNA of 55% (40/73) of CRC patients, but not in 73 adenomas (4%) and 74 subjects without neoplasia (7%) (Pedersen et al., 2014). Complementing this, our study demonstrates that a region of the *QKI* gene (chr6: 163,834,452–163,834,924) has similar robustness in distinguishing CRC from normal tissues and is a CRC-specific methylation marker relative to 30 other types of cancer. Iwata et al. (2017) demonstrated that gene expression of *QKI* was significantly down-regulated in CRC tissues (*p* = 0.049), and patients with low tumor *QKI* expression were associated with worse prognoses (*n* = 153). In contrast, our survival analysis based on the TCGA CRC dataset (*n* = 361) and our in-house validation dataset (*n* = 246) did not suggest a significant association between the methylation level of the *QKI* promoter and the overall survival of CRC (Supplementary Figure S6).

This study has several limitations. First, all samples in the validation dataset were collected from a single center. Second, although our study demonstrated that the *QKI* gene was hypermethylated in cfDNA samples from CRC patients, its clinical utility should be further evaluated in cohorts with more cfDNA samples.

## Conclusion

By integrating genome-wide methylation data from tissues and cell lines of multiple cancer types, this study revealed that methylation of the *QKI* promoter was a CRC-specific marker. Its diagnostic performance is stable in patients with CRC and its precursor lesions from the GEO database and our in-house dataset. Hypermethylation in cfDNA of CRC patients indicates that *QKI* methylation is a tissue-specific and non-invasive marker for the detection of CRC, and is worthy of further verification in a large-scale cohort.

## Data Availability

The datasets presented in this study can be found in online repositories. The names of the repository/repositories and accession number(s) can be found at: https://doi.org/10.6084/m9.figshare.20055134.

## Footnotes

1
https://xena.ucsc.edu/public/.
