# Supplementary material for: Promoter Methylation of QKI as a Potential Specific Biomarker for Early Detection of Colorectal Cancer
Source: Front Genet. 2022 Aug 9;13:928150. doi: 10.3389/fgene.2022.928150 (PMC9395658; doi:10.3389/fgene.2022.928150)
Supplement: Supplementary file 1 [file Table1.DOCX]

Supplementary Material

## Supplementary Figures


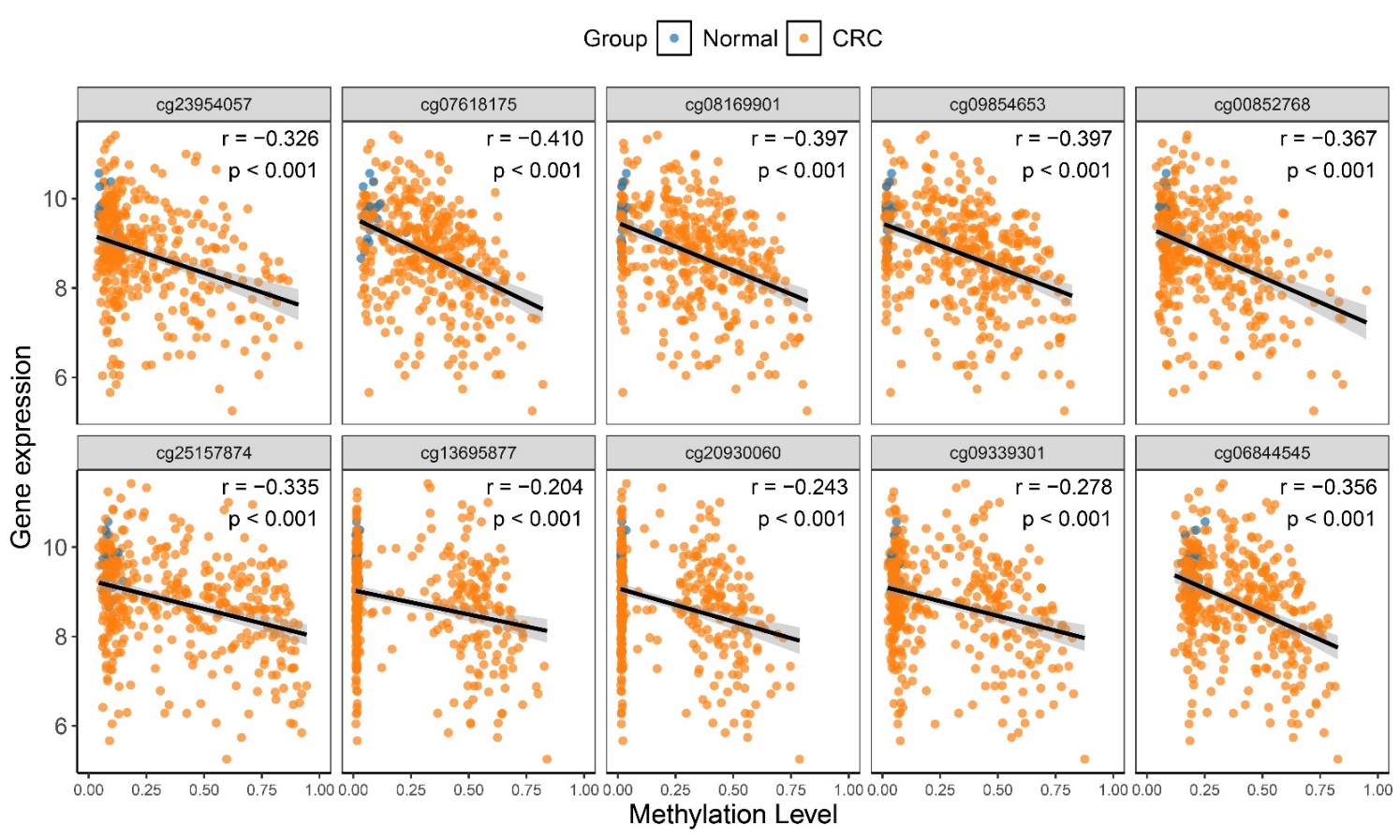
**Supplementary Figure 1.** Spearman correlation analysis between methylation levels of 10 CpG sites at *QKI* promoter and gene expression in the TCGA CRC dataset.


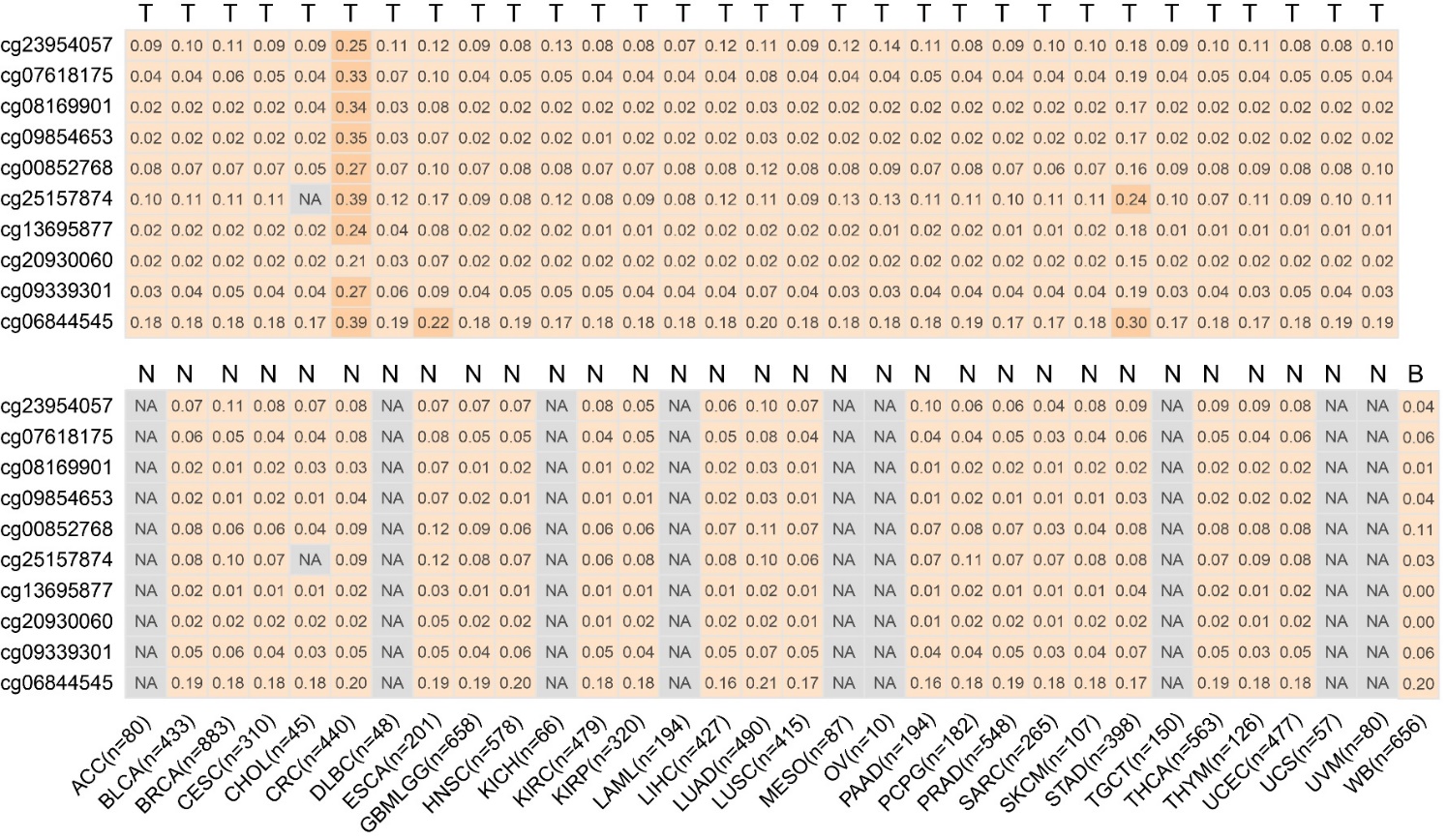


**Supplementary Figure 2.** The average methylation level of 10 CpG sites at *QKI* promoter in 31 cancer and adjacent normal tissues in the discovery dataset. The horizontal axis is the type of cancers and the total number of samples. T: Tumor tissue; N: Normal tissue; B: Whole blood.


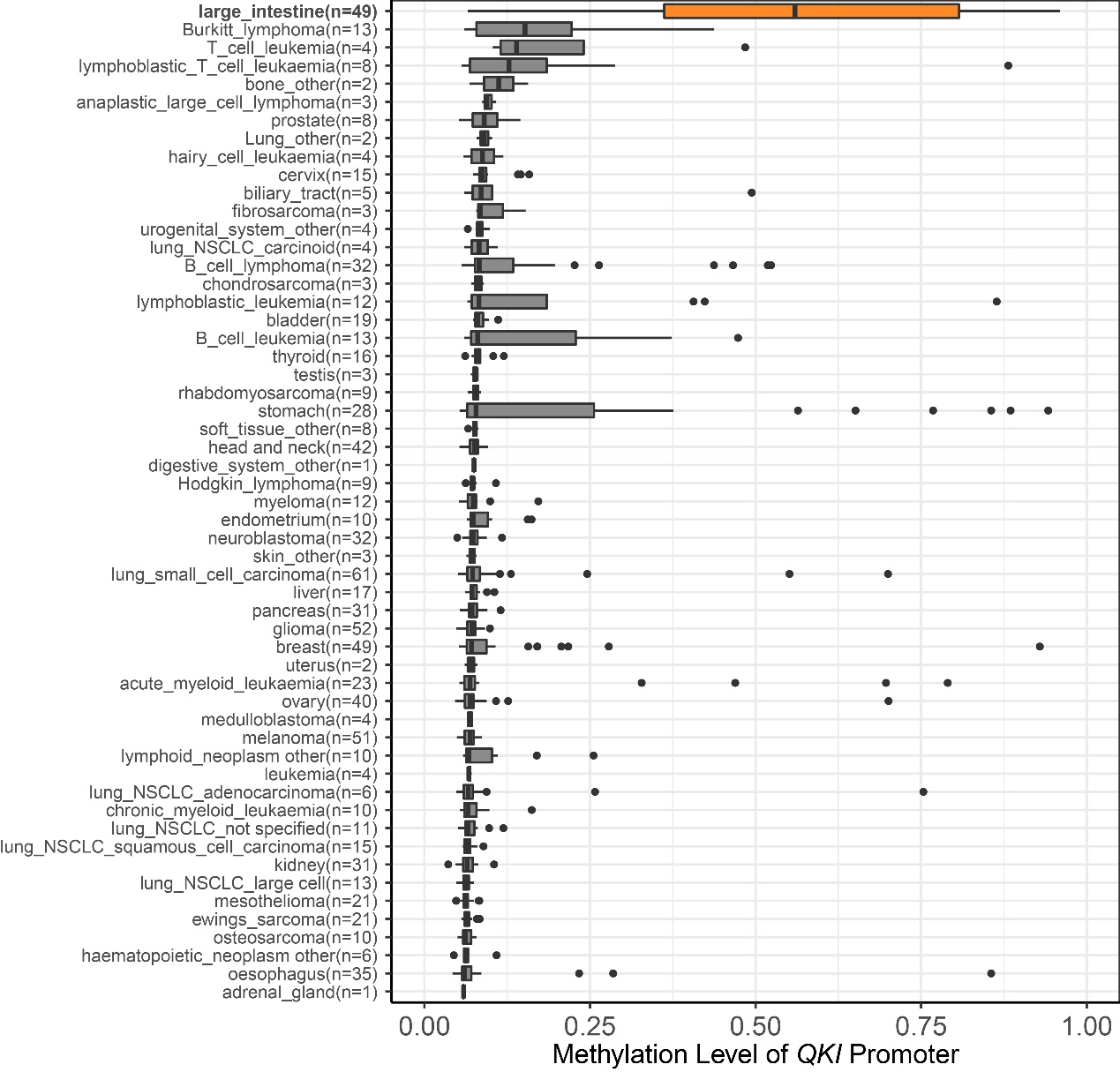


**Supplementary Figure 3.** The average methylation level of the *QKI* promoter in multiple cancer cell lines in the discovery dataset.


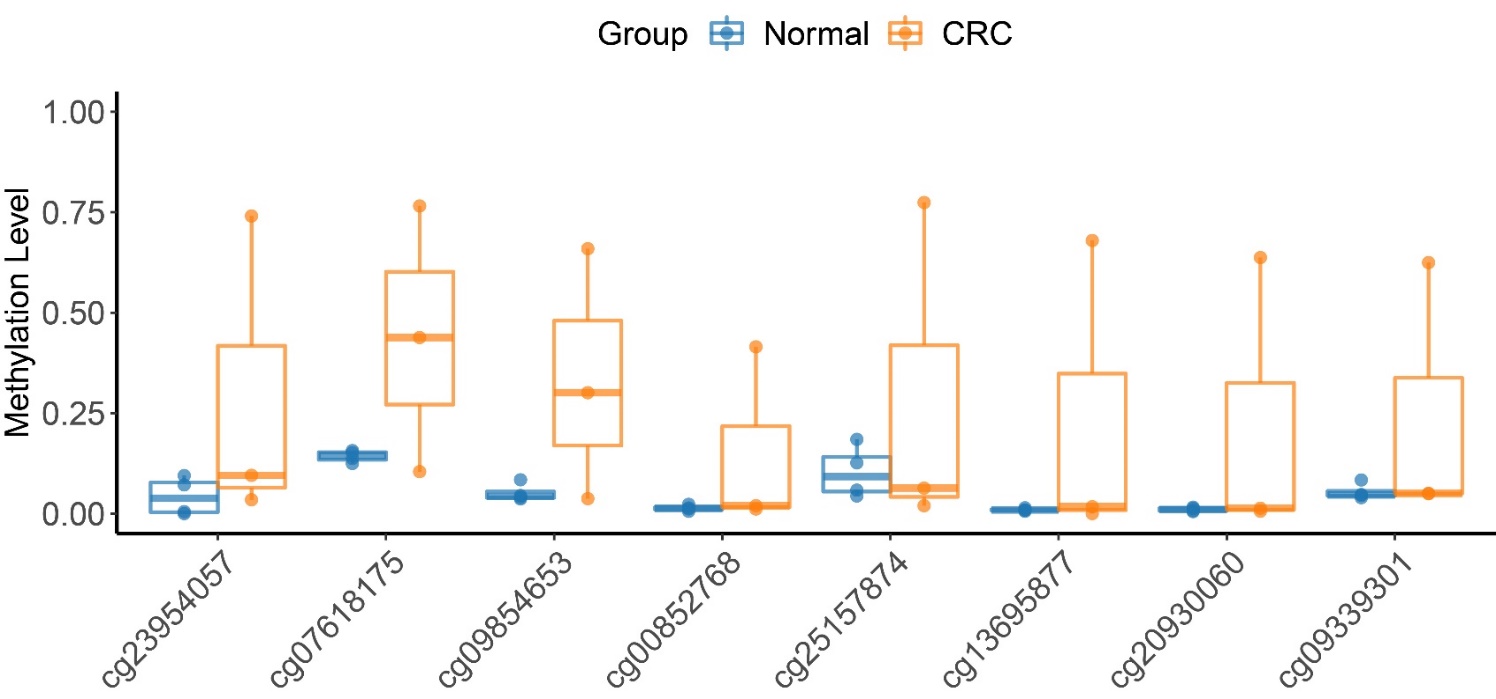


**Supplementary Figure 4.** Methylation status of 8 CpG sites at *QKI* promoter in cell-free DNA samples from 3 colorectal cancer patients and 4 healthy controls in the test set C.


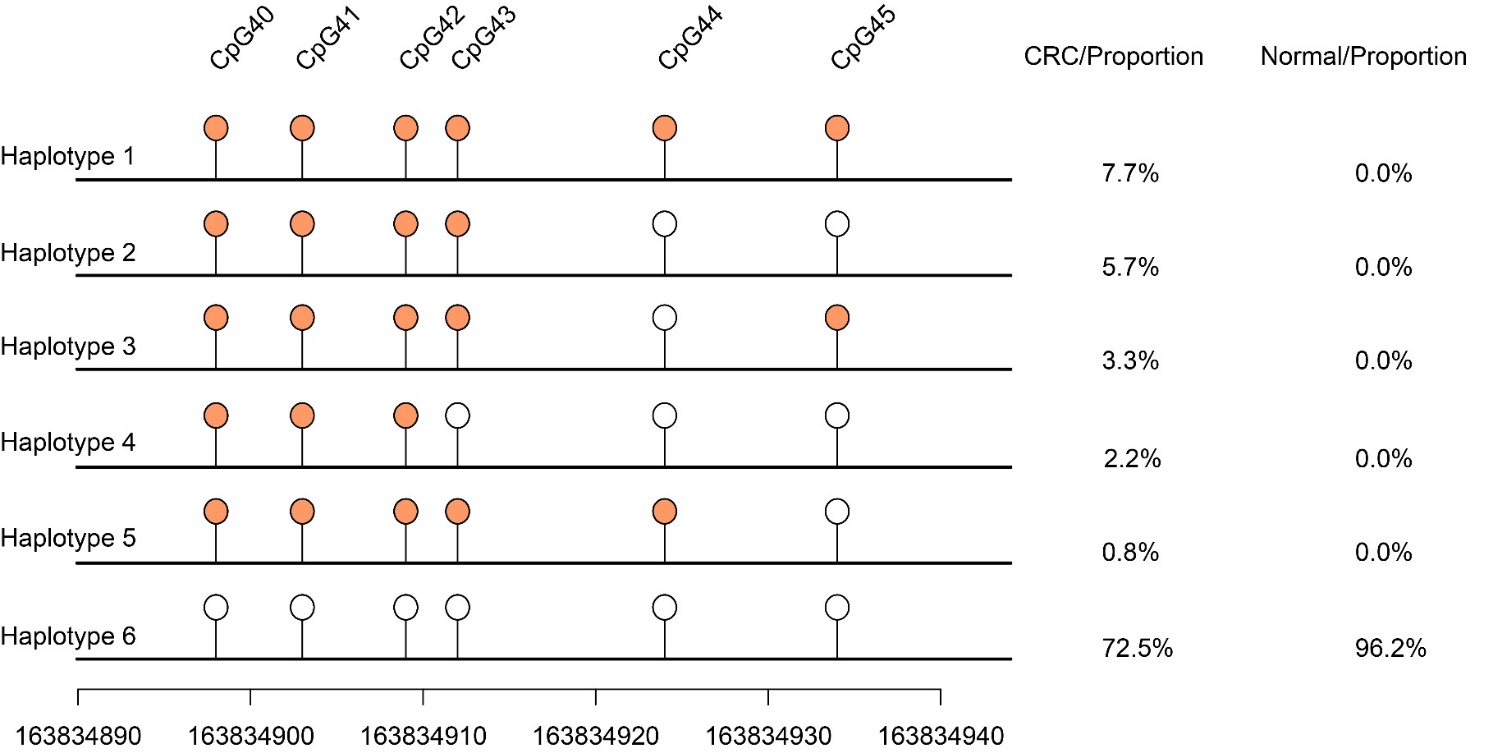


**Supplementary Figure 5.** Methylation haplotypes composed of 6 CpG sites and the proportion in cfDNA samples of CRC and healthy controls. The orange is the methylated CpG sites, and the white is unmethylated CpG sites. We showed the top 6 methylation haplotypes that account for a relatively high proportion.


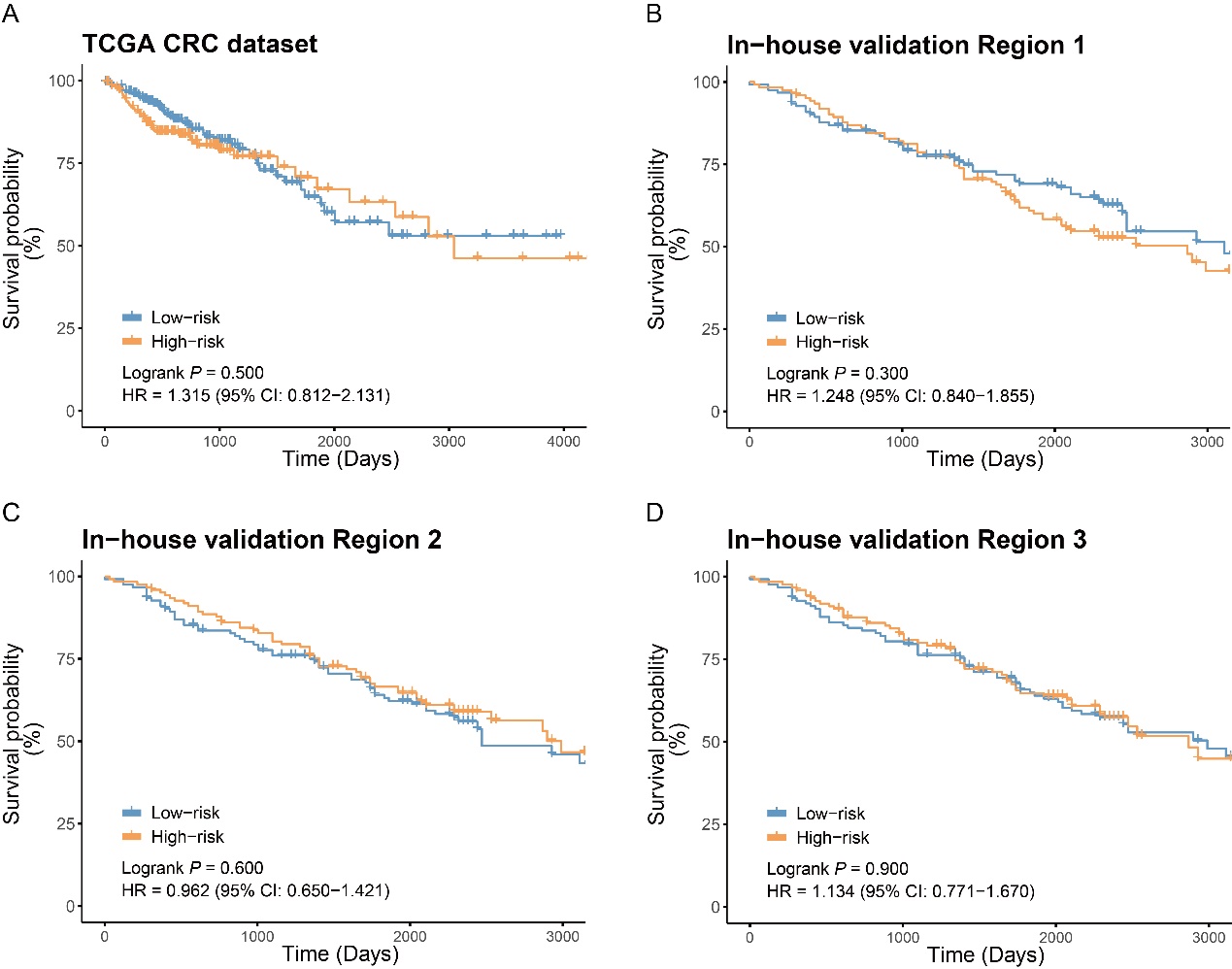


**Supplementary Figure 6.** Association between average methylation level of *QKI* promoter and overall survival of patients with colorectal cancer from (A) the TCGA CRC dataset and (B-D) the validation dataset. The median methylation level was regarded as the cutoff point.

## Supplementary Tables

Supplementary Table S1. Genome information of 25 CpG sites of *QKI* gene in Infinium 450K array.

| CpG Site | Chromosome | Genomic Coordinate | Strand | Relation  to Island | UCSC  RefGene Group |
| --- | --- | --- | --- | --- | --- |
| cg23954057 | chr6 | 163834176 | + | OpenSea | TSS1500 |
| cg07618175 | chr6 | 163834452 | + | OpenSea | TSS1500 |
| cg08169901 | chr6 | 163834872 | + | OpenSea | TSS1500 |
| cg09854653 | chr6 | 163834903 | + | OpenSea | TSS1500 |
| cg00852768 | chr6 | 163834924 | + | OpenSea | TSS1500 |
| cg25157874 | chr6 | 163835117 | + | OpenSea | TSS1500 |
| cg13695877 | chr6 | 163835251 | - | OpenSea | TSS1500 |
| cg20930060 | chr6 | 163835395 | + | OpenSea | TSS1500 |
| cg00931179^†^ | chr6 | 163835582 | + | OpenSea | TSS200 |
| cg09339301 | chr6 | 163836245 | + | OpenSea | 1stExon |
| cg06844545 | chr6 | 163836348 | - | OpenSea | 1stExon |
| cg24583770 | chr6 | 163836718 | - | OpenSea | Body |
| cg05850377 | chr6 | 163837794 | + | OpenSea | Body |
| cg05329960 | chr6 | 163845084 | - | OpenSea | Body |
| cg26728382 | chr6 | 163848720 | + | OpenSea | Body |
| cg17736997 | chr6 | 163851615 | + | OpenSea | Body |
| cg15622891 | chr6 | 163862445 | + | OpenSea | Body |
| cg00254888 | chr6 | 163880196 | - | OpenSea | Body |
| cg14090920 | chr6 | 163911136 | + | OpenSea | Body |
| cg18052967 | chr6 | 163951256 | + | OpenSea | Body |
| cg15542924^†^ | chr6 | 163959346 | + | OpenSea | Body |
| cg07980324^†^ | chr6 | 163965269 | + | OpenSea | Body |
| cg24310785 | chr6 | 163985223 | + | OpenSea | Body |
| cg19630242 | chr6 | 163987817 | + | OpenSea | Body |
| cg21024969 | chr6 | 163994355 | + | OpenSea | 3'UTR |

Note: ^†^ The CpG sites with a missing value above 10% in the TCGA CRC dataset.

Supplementary Table S2. Clinical information on tissue and white blood cell samples.

|  | CRC (Tissue) | Normal (Tissue) | CRC (WBC) | Healthy control (WBC) |
| --- | --- | --- | --- | --- |
| Number | 275 | 24 | 29 | 29 |
| Age | 59.0 ± 10.3 | 59.1 ± 7.1 | 58.1 ± 9.2 | 58.5 ± 9.2 |
| Gender |  |  |  |  |
| Male | 171 (62.2%) | 19 (79.2%) | 22 (75.9%) | 22 (75.9%) |
| Female | 104 (37.8%) | 5 (20.8%) | 7 (24.1%) | 7 (24.1%) |
| Tumor location |  |  |  |  |
| Colon | 97 (35.3%) | - | - | - |
| Rectum | 178 (64.7%) | - | - | - |
| Tumor stage |  |  |  |  |
| I | 22 (8.0%) | - | - | - |
| II | 123 (44.7%) | - | - | - |
| III | 115 (41.8%) | - | - | - |
| IV | 11 (4.0%) | - | - | - |
| Not reported | 4 (1.5%) | - | - | - |

Supplementary Table S3. The primers of regions of *QKI* in targeted bisulfite sequencing.

| Sample Type | Primer | Primer Sequence (5'-3') | Genomic Position ^†^ | Amplicon Length (bp) | Strand | Number of CpGs |
| --- | --- | --- | --- | --- | --- | --- |
| Tissues & WBCs | Forward | GGAGGGTTGGTGGGTTTTT | chr6:163834898-163834632 | 267 | - | 39 |
|  | Reverse | TTCCAACCTCTCAACAAATCAC |  |  |  |  |
|  | Forward | GGGTTGGGTTTGGTYGTTTAG | chr6:163834949-163834791 | 159 | - | 15 |
|  | Reverse | CRAAAAACCTCAAACTCTTATCCTC |  |  |  |  |
|  | Forward | GTATYGTGGGYGAYGGGTTAAGAT | chr6:163834899-163835160 | 262 | + | 33 |
|  | Reverse | AACRTCCTATTACTTCACRAAAAACTTTC |  |  |  |  |
| cfDNA | Forward | GGTTYGAYGGTTAAGGAAAGTTTATTA | chr6:163834865-163834963 | 99 | + | 6 |
|  | Reverse | CRACAACCRACTAAAAACTAAATC |  |  |  |  |

Note: ^†^ The genomic positions based on the GRCh37/hg19 coordinates.

Supplementary Table S4. Differential methylation analyses of 22 available CpG sites in the *QKI* gene between colorectal cancer and adjacent normal tissues in the TCGA discovery dataset.

| CpG Site | UCSC  RefGene Group | Mean CRC | Mean Normal | Difference | *P-*value |
| --- | --- | --- | --- | --- | --- |
| cg23954057 | TSS1500 | 0.253 | 0.079 | 0.174 | 4.21E-45 |
| cg07618175 | TSS1500 | 0.331 | 0.077 | 0.254 | 1.40E-79 |
| cg08169901 | TSS1500 | 0.338 | 0.026 | 0.312 | 1.41E-100 |
| cg09854653 | TSS1500 | 0.354 | 0.039 | 0.314 | 2.88E-82 |
| cg00852768 | TSS1500 | 0.270 | 0.091 | 0.179 | 1.46E-51 |
| cg25157874 | TSS1500 | 0.388 | 0.096 | 0.292 | 4.57E-61 |
| cg13695877 | TSS1500 | 0.241 | 0.016 | 0.225 | 4.74E-48 |
| cg20930060 | TSS1500 | 0.208 | 0.018 | 0.191 | 8.47E-49 |
| cg09339301 | 1stExon | 0.266 | 0.051 | 0.215 | 1.07E-51 |
| cg06844545 | 1stExon | 0.388 | 0.204 | 0.184 | 2.52E-51 |
| cg24583770 | Body | 0.508 | 0.350 | 0.158 | 2.06E-15 |
| cg05850377 | Body | 0.178 | 0.041 | 0.137 | 7.67E-37 |
| cg05329960 | Body | 0.765 | 0.841 | -0.075 | 2.06E-09 |
| cg26728382 | Body | 0.710 | 0.655 | 0.055 | 0.001 |
| cg17736997 | Body | 0.748 | 0.827 | -0.078 | 7.38E-12 |
| cg15622891 | Body | 0.536 | 0.878 | -0.342 | 6.09E-91 |
| cg00254888 | Body | 0.923 | 0.939 | -0.015 | 2.99E-05 |
| cg14090920 | Body | 0.746 | 0.926 | -0.181 | 2.43E-49 |
| cg18052967 | Body | 0.801 | 0.868 | -0.067 | 2.72E-06 |
| cg24310785 | Body | 0.887 | 0.924 | -0.037 | 8.46E-20 |
| cg19630242 | Body | 0.933 | 0.942 | -0.009 | 0.005 |
| cg21024969 | 3'UTR | 0.838 | 0.926 | -0.088 | 8.11E-24 |

Supplementary Table S5. Receiver operating characteristic curve analyses of 39 CpG sites in target region 1 for distinguishing colorectal cancer from adjacent normal tissues in the validation dataset.

| CpG Site | Genomic Coordinate | AUC | Cut-off Value | Sensitivity (%) | Specificity (%) |
| --- | --- | --- | --- | --- | --- |
| CpG1 | 163834653 | 0.904 | 0.051 | 86.3 | 95.8 |
| CpG2 | 163834660 | 0.913 | 0.063 | 86.0 | 95.8 |
| CpG3 | 163834672 | 0.914 | 0.051 | 86.7 | 95.8 |
| CpG4 | 163834675 | 0.914 | 0.055 | 87.1 | 95.8 |
| CpG5 | 163834678 | 0.911 | 0.044 | 87.1 | 95.8 |
| CpG6 | 163834681 | 0.907 | 0.044 | 87.5 | 95.8 |
| CpG7 | 163834690 | 0.907 | 0.058 | 86.3 | 95.8 |
| CpG8 | 163834693 | 0.913 | 0.058 | 86.3 | 95.8 |
| CpG9 | 163834695 | 0.914 | 0.044 | 87.1 | 95.8 |
| CpG10 | 163834697 | 0.909 | 0.046 | 86.3 | 95.8 |
| CpG11 | 163834708 | 0.914 | 0.117 | 81.5 | 100 |
| CpG12 | 163834711 | 0.908 | 0.114 | 81.9 | 100 |
| CpG13 | 163834719 | 0.918 | 0.045 | 87.1 | 95.8 |
| CpG14 | 163834726 | 0.910 | 0.046 | 86.3 | 95.8 |
| CpG15 | 163834730 | 0.904 | 0.052 | 86.0 | 95.8 |
| CpG16 | 163834732 | 0.907 | 0.052 | 86.0 | 95.8 |
| CpG17 | 163834735 | 0.907 | 0.124 | 80.8 | 100 |
| CpG18 | 163834739 | 0.902 | 0.045 | 86.3 | 95.8 |
| CpG19 | 163834745 | 0.906 | 0.062 | 84.9 | 95.8 |
| CpG20 | 163834749 | 0.903 | 0.059 | 85.6 | 95.8 |
| CpG21 | 163834752 | 0.917 | 0.062 | 86.0 | 95.8 |
| CpG22 | 163834762 | 0.905 | 0.056 | 85.6 | 95.8 |
| CpG23 | 163834770 | 0.910 | 0.055 | 86.0 | 95.8 |
| CpG24 | 163834774 | 0.907 | 0.039 | 86.3 | 95.8 |
| CpG25 | 163834777 | 0.911 | 0.051 | 86.0 | 95.8 |
| CpG26 | 163834779 | 0.914 | 0.052 | 86.3 | 95.8 |
| CpG27 | 163834784 | 0.905 | 0.040 | 86.3 | 95.8 |
| CpG28 | 163834786 | 0.912 | 0.048 | 86.0 | 95.8 |
| CpG29 | 163834791 | 0.907 | 0.059 | 86.0 | 95.8 |
| CpG30 | 163834815 | 0.899 | 0.035 | 83.4 | 95.8 |
| CpG31 | 163834824 | 0.910 | 0.043 | 85.6 | 95.8 |
| CpG32 | 163834835 | 0.913 | 0.059 | 86.0 | 95.8 |
| CpG33 | 163834840 | 0.913 | 0.101 | 81.9 | 100 |
| CpG34 | 163834853 | 0.905 | 0.040 | 86.0 | 95.8 |
| CpG35 | 163834855 | 0.909 | 0.039 | 86.3 | 95.8 |
| CpG36 | 163834858 | 0.907 | 0.042 | 86.7 | 95.8 |
| CpG37 | 163834863 | 0.909 | 0.040 | 86.3 | 95.8 |
| CpG38 | 163834869 | 0.905 | 0.055 | 84.9 | 95.8 |
| CpG39^†^ | 163834872 | 0.896 | 0.040 | 84.9 | 95.8 |

Note: ^†^ The CpG39 was cg08169901 in the annotation information of Infinium 450K array.

Supplementary Table S6. Receiver operating characteristic curve analyses of 15 CpG sites in target region 2 for distinguishing colorectal cancer from adjacent normal tissues in the validation dataset.

| CpG Site | Genomic Coordinate | AUC | Cut-off Value | Sensitivity (%) | Specificity (%) |
| --- | --- | --- | --- | --- | --- |
| CpG30 | 163834815 | 0.907 | 0.053 | 83.4 | 95.8 |
| CpG31 | 163834824 | 0.914 | 0.066 | 86.0 | 95.8 |
| CpG32 | 163834835 | 0.917 | 0.193 | 81.9 | 100 |
| CpG33 | 163834840 | 0.914 | 0.143 | 84.1 | 100 |
| CpG34 | 163834853 | 0.904 | 0.063 | 85.6 | 95.8 |
| CpG35 | 163834855 | 0.909 | 0.062 | 86.0 | 95.8 |
| CpG36 | 163834858 | 0.904 | 0.085 | 85.2 | 95.8 |
| CpG37 | 163834863 | 0.908 | 0.074 | 85.6 | 95.8 |
| CpG38 | 163834869 | 0.899 | 0.083 | 84.5 | 95.8 |
| CpG39^†^ | 163834872 | 0.911 | 0.072 | 84.5 | 95.8 |
| CpG40 | 163834898 | 0.898 | 0.128 | 80.8 | 100 |
| CpG41^†^ | 163834903 | 0.895 | 0.068 | 82.3 | 95.8 |
| CpG42 | 163834909 | 0.885 | 0.076 | 82.7 | 95.8 |
| CpG43 | 163834912 | 0.901 | 0.054 | 80.4 | 100 |
| CpG44^†^ | 163834924 | 0.872 | 0.022 | 77.9 | 91.7 |

Note: ^†^ The CpG39, CpG41 and CpG44 were cg08169901, cg09854653 and cg00852768 in the annotation information of Infinium 450K array.

Supplementary Table S7. Receiver operating characteristic curve analyses of 33 CpG sites in target region 3 for distinguishing colorectal cancer from adjacent normal tissues in the validation dataset.

| CpG Site | Genomic Coordinate | AUC | Cut-off Value | Sensitivity (%) | Specificity (%) |
| --- | --- | --- | --- | --- | --- |
| CpG44† | 163834924 | 0.867 | 0.020 | 77.9 | 91.7 |
| CpG45 | 163834934 | 0.861 | 0.104 | 63.8 | 100 |
| CpG46 | 163834955 | 0.826 | 0.164 | 64.9 | 100 |
| CpG47 | 163834962 | 0.842 | 0.169 | 64.6 | 100 |
| CpG48 | 163834968 | 0.828 | 0.188 | 66.1 | 100 |
| CpG49 | 163834975 | 0.825 | 0.162 | 64.9 | 100 |
| CpG50 | 163834981 | 0.831 | 0.200 | 63.5 | 100 |
| CpG51 | 163834983 | 0.814 | 0.161 | 64.2 | 100 |
| CpG52 | 163834988 | 0.832 | 0.197 | 66.4 | 100 |
| CpG53 | 163835009 | 0.807 | 0.119 | 63.8 | 100 |
| CpG54 | 163835011 | 0.793 | 0.168 | 62.4 | 100 |
| CpG55 | 163835013 | 0.810 | 0.096 | 66.4 | 95.8 |
| CpG56 | 163835017 | 0.807 | 0.026 | 71.2 | 83.3 |
| CpG57 | 163835019 | 0.817 | 0.028 | 67.9 | 87.5 |
| CpG58 | 163835026 | 0.813 | 0.097 | 58.7 | 95.8 |
| CpG59 | 163835029 | 0.775 | 0.096 | 53.9 | 95.8 |
| CpG60 | 163835035 | 0.806 | 0.208 | 59.4 | 100 |
| CpG61 | 163835040 | 0.768 | 0.034 | 65.3 | 87.5 |
| CpG62 | 163835047 | 0.776 | 0.159 | 58.3 | 100 |
| CpG63 | 163835056 | 0.769 | 0.103 | 56.8 | 100 |
| CpG64 | 163835061 | 0.757 | 0.086 | 53.9 | 100 |
| CpG65 | 163835065 | 0.811 | 0.153 | 65.3 | 95.8 |
| CpG66 | 163835071 | 0.789 | 0.143 | 61.3 | 100 |
| CpG67 | 163835074 | 0.822 | 0.189 | 63.5 | 100 |
| CpG68 | 163835076 | 0.803 | 0.146 | 66.1 | 100 |
| CpG69 | 163835078 | 0.794 | 0.189 | 63.5 | 100 |
| CpG70 | 163835082 | 0.825 | 0.213 | 65.3 | 100 |
| CpG71 | 163835084 | 0.814 | 0.256 | 65.3 | 100 |
| CpG72 | 163835099 | 0.780 | 0.211 | 59.4 | 100 |
| CpG73 | 163835105 | 0.810 | 0.178 | 64.2 | 100 |
| CpG74 | 163835110 | 0.824 | 0.185 | 64.9 | 100 |
| CpG75† | 163835117 | 0.782 | 0.184 | 60.5 | 100 |
| CpG76 | 163835131 | 0.809 | 0.166 | 56.8 | 100 |

Note: † The CpG44 and CpG75 were cg00852768 and cg25157874 in the annotation information of Infinium 450K array.

Supplementary Table S8. Differential methylation analysis of 39 CpG sites in target region 1 between WBC samples from CRC patients and healthy controls in the validation dataset.

| CpG Site | Genomic Coordinate | CRC Mean | Normal Mean | Difference | P-value |
| --- | --- | --- | --- | --- | --- |
| CpG1 | 163834653 | 0.005 | 0.008 | -0.003 | 0.004 |
| CpG2 | 163834660 | 0.005 | 0.007 | -0.002 | 0.106 |
| CpG3 | 163834672 | 0.005 | 0.009 | -0.004 | 0.005 |
| CpG4 | 163834675 | 0.005 | 0.006 | -0.001 | 0.071 |
| CpG5 | 163834678 | 0.005 | 0.008 | -0.003 | 0.003 |
| CpG6 | 163834681 | 0.005 | 0.006 | -0.001 | 0.090 |
| CpG7 | 163834690 | 0.005 | 0.007 | -0.002 | 0.006 |
| CpG8 | 163834693 | 0.005 | 0.008 | -0.003 | 0.021 |
| CpG9 | 163834695 | 0.005 | 0.008 | -0.003 | 0.016 |
| CpG10 | 163834697 | 0.005 | 0.008 | -0.003 | 0.001 |
| CpG11 | 163834708 | 0.005 | 0.007 | -0.002 | 0.093 |
| CpG12 | 163834711 | 0.005 | 0.008 | -0.003 | 0.003 |
| CpG13 | 163834719 | 0.006 | 0.007 | -0.001 | 0.218 |
| CpG14 | 163834726 | 0.004 | 0.006 | -0.002 | 0.013 |
| CpG15 | 163834730 | 0.005 | 0.006 | -0.001 | 0.177 |
| CpG16 | 163834732 | 0.005 | 0.009 | -0.004 | 0.000 |
| CpG17 | 163834735 | 0.005 | 0.007 | -0.002 | 0.037 |
| CpG18 | 163834739 | 0.004 | 0.007 | -0.003 | 0.016 |
| CpG19 | 163834745 | 0.005 | 0.008 | -0.003 | 0.018 |
| CpG20 | 163834749 | 0.004 | 0.007 | -0.003 | 0.069 |
| CpG21 | 163834752 | 0.005 | 0.007 | -0.002 | 0.069 |
| CpG22 | 163834762 | 0.005 | 0.006 | -0.001 | 0.132 |
| CpG23 | 163834770 | 0.005 | 0.007 | -0.002 | 0.110 |
| CpG24 | 163834774 | 0.005 | 0.007 | -0.002 | 0.025 |
| CpG25 | 163834777 | 0.005 | 0.006 | -0.001 | 0.439 |
| CpG26 | 163834779 | 0.005 | 0.008 | -0.003 | 0.015 |
| CpG27 | 163834784 | 0.005 | 0.008 | -0.003 | 0.000 |
| CpG28 | 163834786 | 0.005 | 0.008 | -0.003 | 0.002 |
| CpG29 | 163834791 | 0.005 | 0.008 | -0.003 | 0.015 |
| CpG30 | 163834815 | 0.005 | 0.008 | -0.003 | 0.045 |
| CpG31 | 163834824 | 0.005 | 0.008 | -0.003 | 0.057 |
| CpG32 | 163834835 | 0.005 | 0.006 | -0.001 | 0.297 |
| CpG33 | 163834840 | 0.004 | 0.007 | -0.003 | 0.010 |
| CpG34 | 163834853 | 0.004 | 0.006 | -0.002 | 0.011 |
| CpG35 | 163834855 | 0.005 | 0.010 | -0.005 | 0.000 |
| CpG36 | 163834858 | 0.004 | 0.007 | -0.003 | 0.012 |
| CpG37 | 163834863 | 0.006 | 0.008 | -0.002 | 0.090 |
| CpG38 | 163834869 | 0.004 | 0.006 | -0.002 | 0.033 |
| CpG39† | 163834872 | 0.005 | 0.007 | -0.002 | 0.160 |

Note: † The CpG39 was cg08169901 in the annotation information of Infinium 450K array.

Supplementary Table S9. Differential methylation analysis of 15 CpG sites in target region 2 between WBC samples from CRC patients and healthy controls in the validation dataset.

| CpG Site | Genomic Coordinate | CRC Mean | Normal Mean | Difference | P-value |
| --- | --- | --- | --- | --- | --- |
| CpG30 | 163834815 | 0.008 | 0.012 | -0.004 | 0.029 |
| CpG31 | 163834824 | 0.009 | 0.012 | -0.003 | 0.046 |
| CpG32 | 163834835 | 0.009 | 0.012 | -0.003 | 0.103 |
| CpG33 | 163834840 | 0.009 | 0.012 | -0.003 | 0.294 |
| CpG34 | 163834853 | 0.008 | 0.011 | -0.003 | 0.040 |
| CpG35 | 163834855 | 0.009 | 0.011 | -0.002 | 0.105 |
| CpG36 | 163834858 | 0.008 | 0.011 | -0.003 | 0.064 |
| CpG37 | 163834863 | 0.009 | 0.011 | -0.002 | 0.146 |
| CpG38 | 163834869 | 0.007 | 0.010 | -0.003 | 0.091 |
| CpG39† | 163834872 | 0.008 | 0.011 | -0.003 | 0.132 |
| CpG40 | 163834898 | 0.008 | 0.010 | -0.002 | 0.229 |
| CpG41† | 163834903 | 0.007 | 0.010 | -0.003 | 0.122 |
| CpG42 | 163834909 | 0.008 | 0.009 | -0.001 | 0.209 |
| CpG43 | 163834912 | 0.007 | 0.010 | -0.003 | 0.051 |
| CpG44† | 163834924 | 0.006 | 0.008 | -0.002 | 0.039 |

Note: † The CpG39, CpG41, and CpG44 were cg08169901, cg09854653, and cg00852768 in the annotation information of Infinium 450K array.

Supplementary Table S10. Differential methylation analysis of 33 CpG sites in target region 3 between WBC samples from CRC patients and healthy controls in the validation dataset.

| CpG Site | Genomic Coordinate | CRC Mean | Normal Mean | Difference | P-value |
| --- | --- | --- | --- | --- | --- |
| CpG44† | 163834924 | 0.004 | 0.008 | -0.004 | 0.003 |
| CpG45 | 163834934 | 0.003 | 0.004 | -0.001 | 0.093 |
| CpG46 | 163834955 | 0.004 | 0.007 | -0.003 | 0.037 |
| CpG47 | 163834962 | 0.004 | 0.007 | -0.003 | 0.026 |
| CpG48 | 163834968 | 0.004 | 0.007 | -0.003 | 0.057 |
| CpG49 | 163834975 | 0.004 | 0.008 | -0.004 | 0.004 |
| CpG50 | 163834981 | 0.006 | 0.008 | -0.002 | 0.016 |
| CpG51 | 163834983 | 0.004 | 0.009 | -0.005 | 0.002 |
| CpG52 | 163834988 | 0.004 | 0.005 | -0.001 | 0.436 |
| CpG53 | 163835009 | 0.005 | 0.007 | -0.002 | 0.134 |
| CpG54 | 163835011 | 0.005 | 0.007 | -0.002 | 0.216 |
| CpG55 | 163835013 | 0.005 | 0.008 | -0.003 | 0.024 |
| CpG56 | 163835017 | 0.005 | 0.006 | -0.001 | 0.248 |
| CpG57 | 163835019 | 0.004 | 0.008 | -0.004 | 0.051 |
| CpG58 | 163835026 | 0.006 | 0.006 | 0.000 | 0.986 |
| CpG59 | 163835029 | 0.004 | 0.007 | -0.003 | 0.015 |
| CpG60 | 163835035 | 0.006 | 0.007 | -0.001 | 0.536 |
| CpG61 | 163835040 | 0.005 | 0.009 | -0.004 | 0.020 |
| CpG62 | 163835047 | 0.005 | 0.006 | -0.001 | 0.275 |
| CpG63 | 163835056 | 0.005 | 0.008 | -0.003 | 0.014 |
| CpG64 | 163835061 | 0.005 | 0.011 | -0.006 | 0.091 |
| CpG65 | 163835065 | 0.004 | 0.007 | -0.003 | 0.023 |
| CpG66 | 163835071 | 0.005 | 0.007 | -0.002 | 0.231 |
| CpG67 | 163835074 | 0.004 | 0.007 | -0.003 | 0.014 |
| CpG68 | 163835076 | 0.005 | 0.008 | -0.003 | 0.048 |
| CpG69 | 163835078 | 0.005 | 0.006 | -0.001 | 0.547 |
| CpG70 | 163835082 | 0.006 | 0.008 | -0.002 | 0.435 |
| CpG71 | 163835084 | 0.005 | 0.006 | -0.001 | 0.594 |
| CpG72 | 163835099 | 0.004 | 0.007 | -0.003 | 0.030 |
| CpG73 | 163835105 | 0.005 | 0.006 | -0.001 | 0.461 |
| CpG74 | 163835110 | 0.003 | 0.009 | -0.006 | 0.027 |
| CpG75† | 163835117 | 0.005 | 0.007 | -0.002 | 0.083 |
| CpG76 | 163835131 | 0.012 | 0.014 | -0.002 | 0.080 |

Note: † The CpG44 and CpG75 were cg00852768 and cg25157874 in the annotation information of Infinium 450K array.

Supplementary Table S11. The average methylation level of the target region in cfDNA samples from CRC and healthy controls.

| CpG Site | Genomic Coordinate | CRC Mean (n = 9) | Normal Mean (n = 5) |
| --- | --- | --- | --- |
| CpG40 | 163834898 | 0.230 | 0.036 |
| CpG41† | 163834903 | 0.222 | 0.018 |
| CpG42 | 163834909 | 0.221 | 0.003 |
| CpG43 | 163834912 | 0.191 | 0.006 |
| CpG44† | 163834924 | 0.106 | 0.007 |
| CpG45 | 163834934 | 0.136 | 0.030 |

Note: † The CpG41 and CpG44 were cg09854653 and cg00852768 in the annotation information of Infinium 450K array.
